# Impaired Corneal Biomechanical Properties and the Prevalence of Keratoconus in Mitral Valve Prolapse

**DOI:** 10.1155/2014/402193

**Published:** 2014-04-17

**Authors:** Emine Kalkan Akcay, Murat Akcay, Betul Seher Uysal, Pinar Kosekahya, Abdullah Nabi Aslan, Mehtap Caglayan, Cemal Koseoglu, Fatma Yulek, Nurullah Cagil

**Affiliations:** ^1^Department of Ophthalmology, Ankara Ataturk Training and Research Hospital, Yildirim Beyazit University, 06810 Ankara, Turkey; ^2^Department of Cardiology, Ankara Ataturk Training and Research Hospital, Yildirim Beyazit University, 06810 Ankara, Turkey

## Abstract

*Objective*. To investigate the biomechanical characteristics of the cornea in patients with mitral valve prolapse (MVP) and the prevalence of keratoconus (KC) in MVP. *Materials and Methods*. Fifty-two patients with MVP, 39 patients with KC, and 45 control individuals were recruited in this study. All the participants underwent ophthalmologic examination, corneal analysis with the Sirius system (CSO), and the corneal biomechanical evaluation with Reichert ocular response analyzer (ORA). *Results*. KC was found in six eyes of four patients (5.7%) and suspect KC in eight eyes of five patients (7.7%) in the MVP group. KC was found in one eye of one patient (1.1%) in the control group (*P* = 0.035). A significant difference occurred in the mean CH and CRF between the MVP and control groups (*P* = 0.006 and *P* = 0.009, resp.). All corneal biomechanical and topographical parameters except IOPcc were significantly different between the KC-MVP groups (*P* < 0.05). *Conclusions*. KC prevalence is higher than control individuals in MVP patients and the biomechanical properties of the cornea are altered in patients with MVP. These findings should be considered when the MVP patients are evaluated before refractive surgery.

## 1. Introduction

Mitral valve prolapse (MVP) is a primary connective tissue abnormality of leaflets, the chordae tendineae, and the annulus of the mitral valves [1]. The prevalence of MVP in the general population ranges from 0.6% to10% and can change according to the diagnostic methods used, the diagnostic criteria, and the population assessed [2]. MVP can be associated with ophthalmological diseases such as keratoconus (KC), chronic progressive external ophthalmoplegia, and retinal artery embolism [3, 4].

KC is a progressive, noninflammatory, idiopathic corneal ectasia characterized by changes in corneal collagen structure and organization [5, 6]. The prevalence of KC in the general population is 50–230 per 100,000 (approximately 1/2000) [7]. It is most commonly an isolated condition, despite the multiple singular reports of coexistence with other disorders [8]. Commonly recognized associations include MVP, Down syndrome, Leber's congenital amaurosis, and various connective tissue disorders [8].

The corneal stroma is the main structure that provides corneal refraction, mechanical properties, and corneal transparency. The anterior stromal part plays especially important role in corneal stability and shape [9]. Aging, corneal pathologies, corneal surgery (e.g., LASIK), and systemic diseases may affect corneal biomechanical characteristics by affecting this stromal structure rich in collagen connective tissue [10–14]. Given the cornea's rich collagen connective tissue, corneal biomechanics may be affected by collagen connective tissue diseases.

Both KC and MVP are noninflammatory conditions, the etiology of which has not yet been clearly identified. In most cases of KC, thinning and ectasia of the central cornea with subsequent progressive reduction of vision occurs [15]. Furthermore, both diseases are associated with systemic collagen diseases such as pseudoxanthoma elasticum, Marfan syndrome, Ehlers-Danlos syndrome, and osteogenesis imperfecta [16–18].

Corneal biomechanical properties can be assessed* in vivo* with an ocular response analyzer (ORA, Reichert Ophthalmic Instruments, Buffalo, NY, USA). An ORA provides measurements of Goldmann-related intraocular pressure (IOPg), corneal-compensated intraocular pressure (IOPcc), corneal hysteresis (CH), and the corneal resistance factor (CRF). CH is related to the viscoelastic property of the cornea. CRF is a parameter that shows the general resistance of the cornea, while IOPcc is a parameter measured according to CH value. The assessment of corneal biomechanics can be used to prevent the misinterpretation of intraocular pressure (IOP) measurements, the preoperative evaluation of patients for refractive surgery, and the separation of healthy and abnormal corneas [12–14, 19, 20].

Despite various studies of MVP prevalence in patients with KC only two studies of KC prevalence in patients with MVP exist [4, 21]. Furthermore, no research on corneal biomechanical characteristics in patients with MVP is available.

This study therefore aimed to compare the biomechanical characteristics of the cornea in MVP patients, as well as to investigate the prevalence of KC in MVP.

## 2. Materials and Methods

This prospective, cross-sectional, comparative study was performed at the Ophthalmology and Cardiology Departments at Yildirim Beyazit University Ankara Ataturk Training and Research Hospital. The study followed the tenets of the Declaration of Helsinki and was approved by the Local Ethics Committee. All participants received oral and written information about the study and, prior to participating, each provided informed, written consent.

Participants were divided into three groups: the MVP group, the KC group, and the control group. The MVP group included 104 eyes of 52 patients, the KC group included 78 eyes of 39 patients, and the control group included 90 eyes of 45 participants.

Transthoracic echocardiography was performed by using a Vingmed System V echocardiography system (GE Vingmed, Horten, Norway) and a 2.5–3.5 MHz transducer in the Cardiology Department. Two investigators performed independent echocardiographic evaluation, and in the case of any discrepancy, consensus was reached between them. Diagnosis of primary MVP was based on the condition that one or both mitral leaflets prolapsed into the left atrium by passing the level of the mitral annulus by at least 2 mm along the parasternal long axis during the systolic phase [22, 23].

Each participant received a complete ophthalmic evaluation that included the assessment of visual acuity, slit-lamp biomicroscopic examination, and fundoscopy. All participants also received a corneal topographical evaluation with a Scheimpflug camera combined with Placido corneal topography (Sirius, version 1.2, CSO, Firenze, Italy), which has been found to be repeatable and reliable [24]. Measurements of simulated keratometry 1 (Sim-K1), simulated keratometry 2 (Sim-K2), apical corneal thickness (ACT), and central corneal thickness (CCT) were taken by an experienced examiner in the Refractive Surgery and KC Unit according to Sirius manufacturer's guidelines. Three measurements were made per eye, and the measurement with the best alignment and fixation was selected for data analysis.

Three ORA measurements were taken in all participants by an experienced clinician. Three high-quality (symmetric, well-defined inward, and outward applanation spike height) measurements were obtained for each eye. In the right eye of each participant, four parameters were obtained: CH, CRF, IOPg, and IOPcc.

All 52 patients of the MVP group had pure MVP (i.e., myxomatous degeneration) and no history of rheumatic heart disease, systemic collagen disease, or any other ocular disease.

Topography findings and clinical examination was used to classify the eye as normal, suspect KC, or KC [25]. The eyes with normal corneal thickness (>505 *μ*m), with no sign of ectasia in anterior and posterior corneal curvature were classified as normal. Suspect KC is defined as abnormal localized steepening of the cornea or an asymmetric bow-tie pattern on corneal topographic examination, a normal-appearing cornea on biomicroscopy, and at least 1 of the following signs: steep keratometric curvature greater than 47.0 D, oblique cylinder greater than 1.50 D, an inferior-superior dioptric asymmetry difference in 1.4 to 1.9 D gradient, central corneal thickness less than 505 *μ*m, and an elevation of the posterior corneal surface [26, 27]. KC was diagnosed as having KC by the topographic pattern (asymmetric bow-tie pattern with or without skewed axes) and at least one KC sign in clinical examination (stromal thinning, conical protrusion of the cornea at the apex, Fleischer ring, Vogt striae, and anterior stromal scar) [28]. Control group included patients who were referred to Ophthalmology Department for refractive surgery.

Exclusion criteria were any history of corneal or intraocular surgery, history of glaucoma, unreliable corneal topography (e.g., corneal scar, history of previous keratitis, and corneal inflammation), or poor cooperation for reliable examination and heart disease other than MVP. Contact lenses had to be removed at least 3 weeks prior to examination in the MVP and control groups. In the KC group, all first examinations of patients were included in the study.

### 2.1. Statistical Data Analysis

Continuous variables were evaluated as mean ± SD in statistical analysis and were compared with Student's *t*-test. Nominal data were analyzed by Pearson's chi-square test, and the correlation between parameters was interpreted with Pearson's correlation coefficient. Any difference was considered to be statistically significant when the *P* value was less than 0.05. Analysis was conducted by using SPSS version 17 (Statistical Package for Social Sciences Inc., Chicago, IL, USA).

## 3. Results

Table 1 shows the baseline characteristics of the MVP, KC, and control groups. Regarding age and gender, no significant differences occurred between the MVP and control groups (*P* = 0.053 and *P* = 0.6, resp.). In the KC group, the mean age was significantly less than that of the MVP and control groups (*P* < 0.001 and *P* < 0.001, resp.).

A significant difference occurred in the mean CH and CRF between the MVP and control groups (*P* = 0.006 and *P* = 0.009, resp.) (Table 2). In the MVP group, mean IOPg, IOPcc, ACT, and CCT measurements were less than those of the control group, though they did not differ significantly (*P* = 0.067, *P* = 0.38, *P* = 0.39, and *P* = 0.26, resp.) (Tables 2 and 3). Moreover, mean Sim-K1 and Sim-K2 did not differ significantly between the MVP and control groups (*P* = 0.61 and *P* = 0.52, resp.). All corneal biomechanical and topographical parameters except IOPcc were significantly different between the KC-MVP groups and the KC-control groups (*P* < 0.05) (Tables 2 and 3). The cut-off value for corneal thickness was 505 *μ*m, as per studies by Fontes et al. [12, 27]. In our study, CCT fell below the cut-off value in 11 of the 52 patients in the MVP group and one of the 45 control group participants in the psychometric analysis (*P* > 0.05).

**Figure 1:**
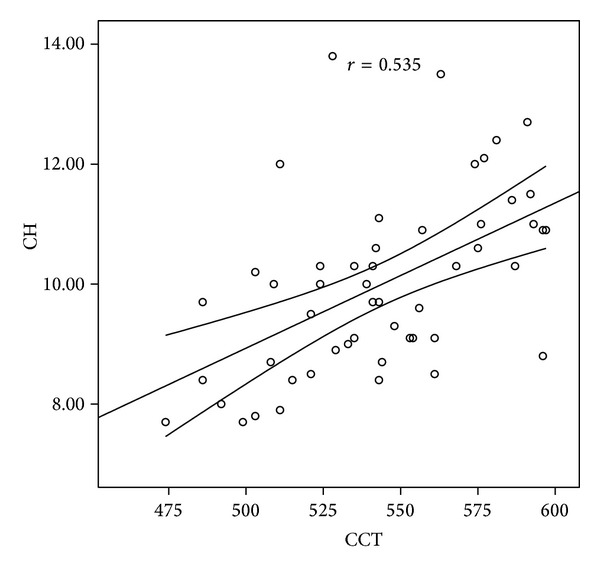
Correlation graphic between CH and CCT. CH: corneal hysteresis, CCT: central corneal thickness.

**Figure 2:**
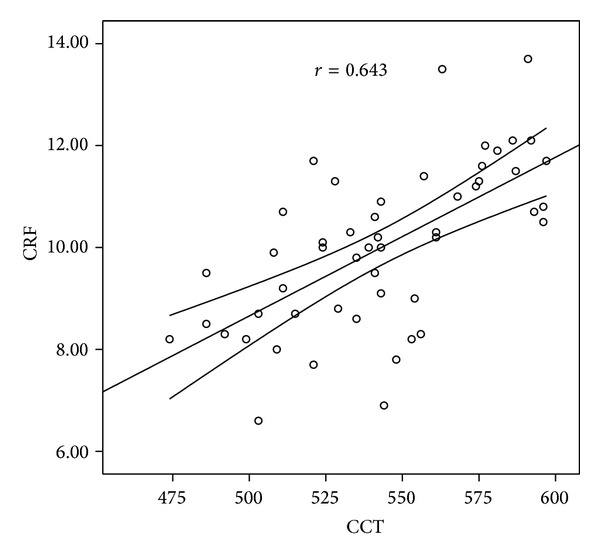
Correlation graphic between CRF and CCT. CRF: corneal resistance factor, CCT: central corneal thickness.

In correlation analysis, we found that in the MVP group, the CH and CRF values were moderately correlated with CCT (*r* = 0.535, *P* = 0.001, and *r* = 0.643, *P* = 0.001, resp.) (Figures 1 and 2). The ACT was also moderately correlated with the CH and CRF values (*r* = 0.462, *P* = 0.001, and *r* = 0.574, *P* = 0.001, resp.).

Furthermore, KC was found in six eyes of four patients (5.7%) and suspect KC in eight eyes of five patients (7.7%) in the MVP group. KC was found in one eye of one patient (1.1%) and suspect KC not found in the control group (*P* = 0.035).

## 4. Discussion

It is well known that human heart valves, like the cornea, are composed of collagen types I and V with a small proportion of type III [29, 30]. Frequently associated with myxomatous degeneration (i.e., the pathological weakening of the connective tissue), MVP is the most common cause of mitral regurgitation and affects 2–22% of the general population [31]. It has been shown that myxomatous degeneration affecting the cornea shares some features with KC; the damage is present particularly in the anterior part of the stroma, Bowman's membrane is disrupted, and stromal keratocytes are transformed into cells with myofibroblastic differentiation [32]. Since the alteration of collagen subtypes occurs in both KC and MVP, it is possible that a single event during embryogenesis affects both structures, for both the corneal stroma and the atrioventricular valves form during the sixth to seventh week of fetal life [9, 16]. Dudakova and Jirsova hypothesized that very similar changes in the extracellular matrix, particularly at the level of collagen metabolism, including lysyl oxidase (LOX) impairment in mitral leaflets, may reflect an association between KC and mitral valve prolapse. As such, these findings may also indicate a very similar origin of KC and MVP [33].

Numerous studies have confirmed a statistically significant higher occurrence of MVP in KC patients, with a reported prevalence of 23–66% compared to the 7–13% prevalence of MVP in the normal population [15, 34, 35].

Though many studies have reported the prevalence of MVP in KC patients, to our knowledge only two studies have reported the prevalence of KC in MVP patients. Lichter et al. studied 72 eyes of 36 MVP patients and 50 eyes of 25 control patients and diagnosed KC in eight eyes of 36 MVP patients (11.1%) and in one (2%) eye of a (4%) patient in the control group [4]. To do so, they used a videokeratography system (Topographic Modeling System, TMS-1, TOMY Computed Anatomy, NY, USA), which is the most sensitive method used in KC diagnosis and also allows early diagnosis. By contrast, Javadi et al. generally used conventional topography in their study but videokeratography (Orbscan, Bausch & Lomb, Rochester, NY, USA) in two patients suspected of having KC yet did not report any KC in 392 MVP patients [21]. In our study, all participants analyzed with a Scheimpflug camera combined with Placido corneal topography, and similar to Lichter et al.'s study, KC and suspect KC prevalence increased (13.4%) in MVP patients compared to those in the control group (1.1%).

Previous studies have found that corneal biomechanical characteristics measured by ORA were affected by numerous corneal diseases, such as KC, Fuchs' endothelial dystrophy, and corneal edema, as well as some systemic factors, including diabetes and menstruation [36, 37]. However, this is the first study conducted to compare the biomechanical properties of the cornea in patients with MVP or KC and a control group.

Our results revealed that, in the MVP group, CH and CRF values were significantly less than those in the control group. These results indicate that corneal viscous and elastic properties between normal and MVP patients differ. To distinguish normal from abnormal corneas before corneal surgery, it is important to assess this biomechanical difference.

Other studies have shown that, in keratoconic eyes, CH and CRF values were less than those in normal eyes [10, 19, 36]. Our results were similar to these; in our study's KC group, values of CH, CRF, IOPcc, and IOPg were less than those in the MVP and control groups. These results indicate that keratoconic eyes are more elastic and less rigid than normal eyes, which emphasizes that the biomechanical characteristics of keratoconic eyes differ from those of normal eyes.

Also in this study, IOPcc measurements did not significantly correlate with CCT, which reveals that IOPcc values provided by ORA seem unaffected by corneal thickness. Similarly, though IOP measurements significantly correlated with CRF, IOPcc values were uninfluenced by CRF, which suggests that IOPcc measurements were also uninfluenced by corneal characteristics.

It is known that keratoconic corneas are thinner, more fragile, and more prone to deformation than normal corneas [27]. We want to underscore that the eyes of MVP patients are similar in some aspects to those of KC patients. Due to structural similarity, corneal thickness in both MVP and KC patients can be less compared to that of normal patients. In our study, CCT was less in 1/5 of MVP patients than in normal individuals (<505 *μ*m). In MVP patients, corneal rigidity and resistance decreased as the cornea thinned [36]. At the same time, ocular resistance was less in MVP patients' eyes due to the combined effect of corneal thickness, ocular rigidity, and characteristics of corneal viscoelasticity. As such, for MVP patients—particularly those diagnosed at an early age—ophthalmologic examination and CCT follow-up are clinically crucial.

Corneal biomechanical characteristics are clinically significant in selecting patients for corneal refractive surgery [19, 38]. Recent reports have suggested that patients with early KC or suspect KC comprise 2–5% of patients who present for refractive surgery for myopia [39, 40].

While evaluating eye problems in MVP patients, especially when planning refractive surgery, corneal biomechanical characteristics and the association of KC with MVP should be considered.

## Figures and Tables

**Table 1:** Demographic characteristics of the patients and the control Group.

Variables	MVP group (*n*: 52)	KC group (*n*: 39)	Control group (*n*: 45)	*P* value	
Age					
Mean ± SD	36.2 ± 12.6	24.8 ± 5.9	32.1 ± 6.6	0.053^a^	0.001^b^
Range	19–65	19–40	21–47		
Gender				0.600^c^	
Female	31 (59.6%)	17 (43.5%)	25 (55.6%)		
Male	21 (40.4%)	22 (56.5%)	20 (44.4%)		

^a^Student's *t*-test, MVP-control group, ^b^Student's *t*- test, MVP-KC group, ^c^Pearson's Chi-Square test, MVP: mitral valve prolapse, KC: keratoconus.

**Table 2:** Corneal biomechanical properties of the patients and the control groups.

Variables	MVP group (*n*: 52)	KC group (*n*: 39)	Control group (*n*: 45)	*P* value^a^	*P* value^b^	*P* value^c^
CH, mmHg	9.46 ± 1.57	7.48 ± 1.35	10.33 ± 1.47	0.006	0.001	0.001
CRF, mmHg	9.51 ± 1.55	6.48 ± 1.78	10.42 ± 1.79	0.009	0.001	0.001
IOPg, mmHg	14.49 ± 3.59	10.38 ± 2.78	15.75 ± 3.59	0.067	0.001	0.001
IOPcc, mmHg	15.44 ± 3.08	14.25 ± 2.35	15.97 ± 2.88	0.38	0.048	0.001

^a^Student's *t*-test, MVP-control group, ^b^Student's *t*-test, MVP-KC group, ^c^Student's *t*-test, KC-control group, MVP: mitral valve prolapse, KC: keratoconus, CH: corneal hysteresis, CRF: corneal resistance factor, IOPg: Goldmann-related intraocular pressure, IOPcc: cornea compensated intraocular pressure.

**Table 3:** Corneal topographical properties of the patients and the control groups.

Variables	MVP group (*n*: 52)	KC group (*n*: 39)	Control group (*n*: 45)	*P* value^a^	*P* value^b^	*P* value^c^
Sim-K1, D	42.92 ± 1.42	47.69 ± 3.34	42.77 ± 1.31	0.61	0.001	0.001
Sim-K2, D	43.97 ± 1.53	52.22 ± 3.86	44.16 ± 1.33	0.52	0.001	0.001
ACT, *μ*m	548.4 ± 31.0	441.8 ± 48.8	554.0 ± 31.9	0.39	0.001	0.001
CCT, *μ*m	539.3 ± 33.4	443.2 ± 42.6	546.69 ± 30.1	0.26	0.001	0.001

^a^Student's *t*-test, MVP-control group, ^b^Student's *t*-test, MVP-KC group, ^c^Student's *t*-test, KC-control group, MVP: mitral valve prolapse, KC: keratoconus, Sim-K1: simulated keratometry 1, Sim-K2: simulated keratometry 2, ACT: apical corneal thickness, CCT: central corneal thickness.
